# Supplementary material for: Untargeted pixel-by-pixel metabolite ratio imaging as a novel tool for biomedical discovery in mass spectrometry imaging
Source: eLife. 2025 Mar 18;13:RP96892. doi: 10.7554/eLife.96892 (PMC11919253; doi:10.7554/eLife.96892)
Supplement: Supplementary file 1. [file elife-96892-supp1.docx]

| **Ratio image tools** | **MSI specific software** | **Workable Image Data Format** | **Targeted input Ratio image** | **Untargeted ratio image** | **Free and open source** |
| --- | --- | --- | --- | --- | --- |
| R workflow | Yes | Excel, imzml | yes | yes | yes |
| ENVI IDL | No | Optical and SAR Data | yes | no | no |
| MATLAB RatioImage App | No | Variety files including Image, Text, Spreadsheet, MATLAB data file | yes | no | no |
| Spectral Python package | No | ENVI, AVIRIS, ERDAS/Lan, imzml | yes | no | yes |
| QGIS hyperspectral image analysis | No | Image, Text, StreetMap, PostGIS etc | yes | no | yes |
