# Supplementary material for: Untargeted pixel-by-pixel metabolite ratio imaging as a novel tool for biomedical discovery in mass spectrometry imaging
Source: eLife. 2025 Mar 18;13:RP96892. doi: 10.7554/eLife.96892 (PMC11919253; doi:10.7554/eLife.96892)
Supplement: Supplementary file 2. [file elife-96892-supp2.pdf]

Metabolite or Metabolite ratio	Glutamine/Glutamate
Glutamine/Glutamate	1
NAA/Glutamine	-0.691412587
Glutamate/Glucose	-0.683182994
Aspartate/Glutamine	-0.617245759
Aspartate/Glucose	-0.571635589
NAA/Glucose	-0.48745363
Glutathione/Glutamine	-0.482699341
Glutamate/Malate	-0.477828273
Glutamate/NAAG	-0.418214673
Glutathione/Glucose	-0.414916374
Glutamate/Taurine	-0.408268788
Aspartate/NAAG	-0.40561461
$FeCl_2$	-0.40137478
