# Supplementary material for: Untargeted pixel-by-pixel metabolite ratio imaging as a novel tool for biomedical discovery in mass spectrometry imaging
Source: eLife. 2025 Mar 18;13:RP96892. doi: 10.7554/eLife.96892 (PMC11919253; doi:10.7554/eLife.96892)
Supplement: Supplementary file 3. [file elife-96892-supp3.docx]

| m/z | Name |
| --- | --- |
| 215.03279 | Glucose+Cl |
| 191.01973 | Citrate |
| 133.01425 | Malate |
| 132.03023 | Aspartate |
| 146.04588 | Glutamate |
| 145.06187 | Glutamine |
| 174.0408 | N-Acetylaspartate |
| 303.08339 | N-Acetylaspartylglutamate |
| 124.00739 | Taurine |
| 306.07653 | Glutathione |
