# Supplementary material for: Untargeted pixel-by-pixel metabolite ratio imaging as a novel tool for biomedical discovery in mass spectrometry imaging
Source: eLife. 2025 Mar 18;13:RP96892. doi: 10.7554/eLife.96892 (PMC11919253; doi:10.7554/eLife.96892)
Supplement: Supplementary file 4. [file elife-96892-supp4.docx]

| m/z | Name |
| --- | --- |
| 478.2939 | LPE 18:1 |
| 506.3252 | LPE 20:1 |
| 500.2782 | LPE 20:4 |
| 528.3095 | LPE 22:4 |
| 524.2782 | LPE 22:6 |
