# Supplementary material for: Untargeted pixel-by-pixel metabolite ratio imaging as a novel tool for biomedical discovery in mass spectrometry imaging
Source: eLife. 2025 Mar 18;13:RP96892. doi: 10.7554/eLife.96892 (PMC11919253; doi:10.7554/eLife.96892)
Supplement: Supplementary file 5. [file elife-96892-supp5.docx]

| Entity Name | Spearman R square (WT) | Spearman R square (KO) |
| --- | --- | --- |
| Glutamate/Glutamine | 1 | 1 |
| Glutamate/Glucose | 0.852795869 | 0.678393963 |
| Glutamate/Aspartic acid | 0.801507737 | 0.558480678 |
| Glutamate/N_Acetylaspartylglutamate | 0.800125626 | 0.597592592 |
| N_Acetylaspartate/N_Acetylaspartylglutamate | 0.759938917 | 0.543231796 |
| Taurine/N_Acetylaspartylglutamate | 0.747279077 | 0.493250032 |
| GSH/N_Acetylaspartylglutamate | 0.725654702 | 0.534683985 |
| Citrate/N_Acetylaspartylglutamate | 0.670060429 | 0.3079266 |
| Aspartate/N_Acetylaspartylglutamate | 0.659178165 | 0.568779025 |
| Glutamate/Malic acid | 0.65035649 | 0.666255195 |
| Taurine/Glutamine | 0.648921859 | 0.554419257 |
| Taurine/Glucose | 0.644297546 | 0.452028615 |
| Glutamine/N_Acetylaspartylglutamate | 0.643225848 | 0.436576981 |
| Malate/N_Acetylaspartylglutamate | 0.624522126 | 0.441813603 |
| Glutamate/N-Acetylaspartate | 0.603496857 | 0.536109488 |
| Glutamate/GSH | 0.592151602 | 0.531586301 |
| Taurine/GSH | 0.567926578 | 0.282270798 |
| Taurine/Malic acid | 0.561595842 | 0.437632948 |
| Glucose/N_Acetylaspartylglutamate | 0.546762829 | 0.456179028 |
| Taurine/Aspartate | 0.543991912 | 0.289845889 |
| N-Acetylaspartate/Glucose | 0.485692311 | 0.460863975 |
| Adenosine | 0.48250312 | 0.339646908 |
| Taurine/N-Acetylaspartate | 0.480117014 | 0.263254553 |
| Taurine/Citrate | 0.444918698 | 0.56298861 |
| Glutamine/Glucose | 0.431762837 | 0.252386832 |
| Glutamate | 0.427270886 | 0.528188618 |
| Glutamate/Citrate | 0.414241714 | 0.592641696 |
| Docosahexaenoic acid/Oleic acid | 0.412424989 | 0.316780465 |
| GSH/Glucose | 0.409754542 | 0.418037354 |
| N-Acetylaspartate/Glutamine | 0.402130321 | 0.503623165 |
| Glutamine/N-Acetylaspartate | -0.402121379 | -0.503623105 |
| Glucose/GSH | -0.409720091 | -0.418038021 |
| Oleic acid/Docosahexaenoic acid | -0.412418152 | -0.316772044 |
| Citrate/Glutamate | -0.41420686 | -0.592642619 |
| Glucose | -0.414832304 | -0.247192996 |
| Glucose/Glutamine | -0.431769617 | -0.252433325 |
| Cytidine | -0.440811865 | -0.091933842 |
| Citrate/Taurine | -0.444846276 | -0.562985142 |
| Glycerylphosphorylethanolamine | -0.446969716 | -0.132695491 |
| N-Acetylaspartate/Taurine | -0.480131641 | -0.263259877 |
| Glucose/Acetylaspartate | -0.485566843 | -0.460873356 |
| Aspartate/Taurine | -0.543994549 | -0.2898528 |
| N_Acetylaspartylglutamate/Glucose | -0.546773877 | -0.456197823 |
| Ribothymidine | -0.553276958 | -0.345023745 |
| Hypoxanthine | -0.55899857 | -0.220084088 |
| Threonate | -0.559486345 | -0.21885935 |
| Malate/Taurine | -0.56159942 | -0.437638439 |
| GSH/Taurine | -0.56793975 | -0.28227912 |
| Glutamylhydroxyproline | -0.576156273 | -0.366588722 |
| GSH/Glutamate | -0.592167807 | -0.531584116 |
| N-Acetylaspartate/Glutamate | -0.603520459 | -0.536111131 |
| N_Acetylaspartylglutamate/Malic acid | -0.624526454 | -0.441825388 |
| N_Acetylaspartylglutamate/Glutamine | -0.643220125 | -0.43659667 |
| Glucose/Taurine | -0.644270344 | -0.452036941 |
| Glutamine/Taurine | -0.64890671 | -0.554426135 |
| Malate/Glutamate | -0.650339314 | -0.666263387 |
| Inosine | -0.652470417 | -0.328246954 |
| **N_Acetylaspartylglutamate** | **-0.657380577** | **-0.463920793** |
| **N_Acetylaspartylglutamate/Aspartic acid** | **-0.659182447** | **-0.568791965** |
| N_Acetylaspartylglutamate/Citrate | -0.670054261 | -0.307934844 |
| N_Acetylaspartylglutamate/GSH | -0.725656919 | -0.53469776 |
| N_Acetylaspartylglutamate/Taurine | -0.747279393 | -0.493256641 |
| **N_Acetylaspartylglutamate/N-Acetylaspartate** | **-0.759938281** | **-0.543230626** |
| N_Acetylaspartylglutamate/Glutamate | -0.800114153 | -0.59760264 |
| Aspartate/Glutamate | -0.80148754 | -0.55847547 |
| Glucose/Glutamate | -0.852793526 | -0.678396625 |
| Glutamine/Glutamate | -0.999998899 | -0.999999747 |
